# Geographic, Socio-Demographic and School Type Variation in Adolescent Wellbeing and Mental Health and Links with Academic Competence in the United Arab Emirates

**DOI:** 10.1007/s12187-022-09993-7

**Published:** 2022-11-23

**Authors:** Jose Marquez, Louise Lambert, Megan Cutts

**Affiliations:** 1 Manchester Institute of Education, University of Manchester, Oxford Road, Manchester, M13 9PL UK; 2 Canadian University Dubai, Dubai, UAE

**Keywords:** Wellbeing, Life Satisfaction, Mental Health, Adolescence, United Arab Emirates

## Abstract

Interest in adolescents’ wellbeing and mental health is growing worldwide, but little research in this area has been conducted in certain world regions and countries such as the United Arab Emirates (UAE). Geographic, socio-demographic and school type differences in adolescent wellbeing and mental health are commonly observed in the field, and the UAE is a diverse country where these types of differences have been found for other outcomes (notably, academic). Yet, no prior national study has explored these differences in terms of wellbeing and mental health in the nation. We address this gap by investigating differences across emirates, gender, socio-economic status, immigrant status, school sector and school curriculum for overall life satisfaction, positive affect, negative affect, meaning and purpose in life, and internalizing difficulties. We use linear regression to analyse cross-sectional data from the Programme for International Student Assessment (PISA) study from 2015 and 2018. We find substantial geographic, socio-demographic and school type differences in levels (2018) of wellbeing and mental health -which vary across distinct domains- and declines (2015–2018) of wellbeing. Better wellbeing and mental health are observed in the northern emirates and among boys. Better wellbeing and poorer mental health are observed among nationals (compared to expatriates) and in public schools (compared to private schools). Despite presenting the best academic outcomes, British schools present the worst wellbeing and mental health outcomes. However, results show the absence of a trade-off between academic competence and wellbeing and mental health, with evidence of a small positive association with wellbeing.

## Introduction

Global attention to the wellbeing and mental health of children and young people has grown (Diener et al., 2018; Thapar et al., 2021). This is partly due to concerns about negative trends observed in many countries over the last two decades (Marquez & Long, 2020; McManus et al., 2019; Mojtabai et al., 2016) and more recent concerns about the impact of the COVID-19 pandemic (Gavin et al., 2021; Racine et al., 2021; UNICEF, 2021).

In the case of wellbeing, interest was originally driven by an increasing global recognition that children have the right to be heard in all aspects affecting their lives (art. 12 UNCRC, United Nations, 1989). This interest was also driven by the need to move beyond “well-becoming” approaches that are concerned with children’s future outcomes and towards wellbeing approaches that consider childhood as a stage of significance in its own right and not as a mere journey to adulthood (Ben-Arieh, 2007).

Still, future outcomes remain a key element motivating interest in child wellbeing, with concerns often focusing on the wellbeing of adolescents. A reason for this is that adolescence has been identified as a window of opportunity for intervention as this is the time when wellbeing begins to decline (Casas and González-Carrasco, 2019) and the prevalence of mental health issues increases (NHS Digital, 2018). Evidence indicates that most lifetime cases of mental ill health manifest by the age of 24, with a peak age of onset of 14.5 years (Colizzi et al., 2020; Solmi et al., 2021). Further evidence indicates that wellbeing in adolescence is predictive of adult wellbeing, mental health, physical health and health behaviour, relational, as well as labour market and socioeconomic outcomes (Cavioni et al., 2021; DeNeve & Oswald, 2012; Goodman et al., 2015; Guzmán et al., 2020; Kansky et al., 2016; Marquez et al., 2022a; Richards & Huppert, 2011). Research on the absence of a trade-off between wellbeing and academic outcome (Gutman & Vorhaus, 2012; Lindorff, 2021; Shoshani et al., 2016) has also helped advanced wellbeing promotion agendas in school contexts.

Wellbeing has traditionally been conceptualised in two ways. In the first, hedonic or subjective wellbeing comprises a cognitive component (i.e., life satisfaction (LS)) and an affective component (i.e., experiencing positive and negative moods, feelings and emotions) (Diener et al., 2002). This view concerns itself with how individuals experience and appraise their own lives. In the second, eudaimonic or psychological wellbeing has been conceptualised in terms of purpose in life, environmental mastery, autonomy, personal growth, self-acceptance, positive relations with others, and optimism (Ryff et al., 2021) and is concerned with how individuals function and relate to others. In sum, although we acknowledge that the term wellbeing/well-being can be used to refer to a range of outcomes in different domains (including also objective measures of well-being such as health status, academic competence, etc.), in this study, we use the term wellbeing to refer to subjective/hedonic and psychological/eudaimonic wellbeing.

Wellbeing is increasingly being studied in combination with mental health in research that, in most cases, defines this in terms of the presence of mental ill-health symptoms of internalizing difficulties (depression, anxiety, social anxiety, somatic complaints, etc.) or, less commonly, externalizing difficulties (under-controlled, impulsive, or aggressive behaviour) (Otto et al., 2021; Petersen et al., 2018; Trotta et al., 2020). Wellbeing and mental health have customarily been conceptualized as two ends of the same spectrum, but research suggests these are independent constructs that, although related, have different correlates and may follow different trajectories over the life course (Bohlmeijer & Westerhof, 2021; Kinderman et al., 2015; Patalay & Fitzsimons, 2016, 2018; Sharpe et al., 2016; Westerhof & Keyes, 2010).

**Fig. 1:**
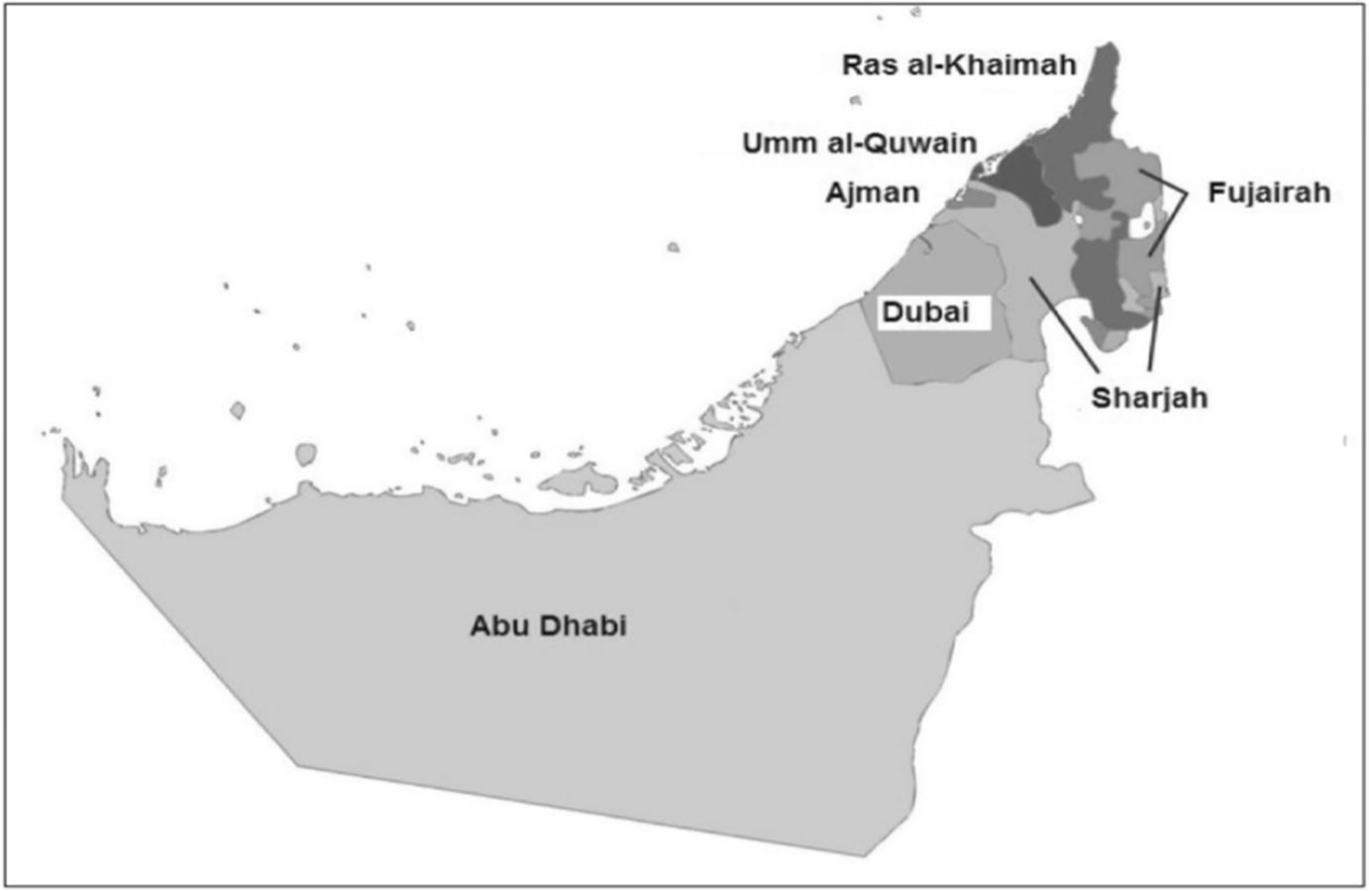
The UAE’s seven emirates

Studying wellbeing and mental health together can provide a more complete picture of the emotional and mental states of individuals. Yet, while much has been learned in the last decades about adolescent wellbeing and mental health in many parts of the world, there is little in others. This is the case of the UAE, a high-income nation in the Gulf Cooperation Council (GCC) region comprised of seven emirates and characterised by a diverse socio-demographic profile (See Fig. 1). The national population represents only around 10% of the nation, who differ from expatriates (around 90%) in terms of linguistic, religious and cultural characteristics (Chan et al., 2021; Maalouf et al., 2019). This diversity is reflected in outcomes for children and young people, including academic outcomes among a student population with almost three in four students enrolled in private schools (Buckner, 2018; Marquez et al., 2022b).

The current study advances our understanding of adolescent wellbeing and mental health inequalities in the UAE across emirates, socio-demographic groups and school types. Such studies are critical in developing and refining education policies, making decisions around resource allocation and responsibilities. This can facilitate that all young people enjoy life with the fewest mental health concerns and the highest wellbeing possible, and that no region, school type or socio-demographic group is left behind. This study focuses on differences in levels of overall LS (OLS), positive affect, negative affect, meaning and purpose in life, and mental health symptoms of internalizing difficulties in 2018, as well as declines in OLS between 2015 and 2018. We also examine the association between academic competence and wellbeing and mental health outcomes.

### Regional, Socio-Demographic and School Type Variation in Adolescent Wellbeing and Mental Health

Research exploring geographic variation in adolescent wellbeing and mental health has grown in recent years. Although there is a large number of studies investigating within-country variation (i.e., Islam et al., 2021; Schoon & Henseke, 2022; Van Phan and O’Brien, 2019), most research in this area focuses on cross-national differences. Evidence from cross-national studies indicate substantial variation across socio-cultural contexts and languages (Campbell et al., 2021; Marquez & Main, 2020; Rees et al., 2015). For instance, in an international study of youth wellbeing across 29 nations, Mutumba and Schulenberg (2020) found that national wellbeing variations were in part explained by place of residence, finding higher subjective wellbeing in rural areas. Urban–rural differences in mental health and wellbeing outcomes have been well documented, with poorer mental health and wellbeing outcomes noted in urban areas (i.e., Buttazzoni et al., 2022; Murphy et al., 2020).

Wellbeing and mental health also vary across socio-demographic groups. For example, there is evidence of wellbeing and mental health inequalities during childhood and adolescence in relation to gender, however, the direction of the inequality shifts throughout development. Boys tend to report higher rates of mental health difficulties than girls in both Western and Middle Eastern countries until puberty (Cyranowski, et al., 2000; NHS Digital, 2021; Patton & Viner, 2007), at which time girls record double the rate until the age of 35 to 40 (Cyranowski et al., 2000; Patalay and Fitzsimons, 2018). In terms of gender differences in adolescent wellbeing, girls tend to present worse outcomes than boys (Bradshaw et al., 2013; Campbell et al., 2021; Kaye-Tzadok et al., 2017), but differences across domains are common. For example, a meta-analysis found that overall LS (OLS) tends to be higher among boys, but satisfaction in some domains is higher among girls (e.g., satisfaction with family and school) and higher among males in others (e.g., satisfaction with time-use, self) (Chen et al., 2019). Using PISA data from 73 nations, Campbell et al. (2021) found that females reported worse outcomes on eudaimonic measures of meaning and purpose in life, psychological distress, OLS, and hedonic measures of positive affect.

Lower socio-economic status (SES) has also been associated with lower LS (i.e., Chzhen et al., 2016; Elgar et al., 2017; Knies, 2022; Marquez, 2021; Patalay & Fitzsimons, 2018; Zaborskis et al., 2019a, b), although how it is measured matters. Associations tend to be more robust in studies using measures of material deprivation compared to family income (Gross-Manos, 2017; Main, 2014; Rees et al., 2011; Sarriera et al., 2014; Zaborskis et al., 2019b) and even more so in studies that use child-derived measures of material well-being compared to those derived by adults (Lau and Bradshaw, 2018; Main, 2014). Researchers have found that children and young people with lower SES also have more mental health issues. A review of 52 studies by Reiss (2013) found a two-to-three-time higher likelihood of developing mental health issues at lower SES levels, with problems rising as SES declined. Meta-analyses (i.e., Peverill et al., 2021; Reiss et al., 2019) have confirmed the same, although to varying degrees and for more granular reasons, i.e., parent education (Merz et al., 2018), parent mental health issues (Amone-P'Olak et al., 2011).

Migration status is another variable associated with wellbeing, although the evidence is mixed. Most find that immigrant adolescents report lower wellbeing than native students (Bradshaw et al., 2011; Casas et al., 2013; Marquez & Main, 2020; Tang, 2019), but others find no differences or even differences in the opposite direction (Filion et al., 2018). Contrasting findings seem to be explained by differences in the host country considered and what aspect of wellbeing is the focus of the study. For example, studies exploring academic and wellbeing outcomes have found that native students have better academic performance, but higher (Wang, 2021) or lower (Rodríguez et al., 2020) scores in positive affect, as well as equal (Alivernini et al., 2020; Rodríguez et al., 2020) or slightly lower (Wang, 2021) scores in LS. Where immigrant adolescents report lower wellbeing, differences are often explained by socio-economic characteristics, as well as friendships and loneliness, discrimination, religiosity, social participation, acculturation, and school related factors (e.g., language, bullying, school-related anxiety, fear of failure, grade repetition, etc.) (Berry & Hou, 2017; Dryden-Peterson, 2018; Lee, 2019; Paparusso, 2021; Schwartz et al., 2013). Differences in LS have been similarly explained: using data from PISA 2015 across 48 countries and economies, Tang, (2019) found that immigrant students reported lower LS, but the gap could be partly explained by factors such as talking to parents, bullying and schoolwork-related anxiety. Research exploring migrant differences in adolescent wellbeing in the UAE is non-existent, and to the best of our knowledge, only one study has examined this for mental health (Shah et al., 2020; described below). Studying this question in the UAE is vital as this is a nation where migrant gaps, particularly in academic outcomes (Marquez et al., 2022b), emerge in the opposite direction compared to most countries.

Finally, wellbeing and mental health differences across types of schools is substantial. This is not surprising considering the role school factors play in explaining variation in wellbeing (Marquez & Main, 2020; Marquez et al., 2022c; Taylor et al., 2022). In an international comparative study using data from PISA 2015, Marquez and Main (2020) found that in 15 of 31 countries, the LS of students differed across types of schools (public, semi-private, private). After controlling for SES and other socio-demographic and school characteristics, LS was higher in private or semi-private schools in six countries and lower in nine countries, suggesting that the wellbeing impact of attending private schools may differ by country. Studies in the UK have found that private schools offer no mental health and wellbeing advantages (Henderson et al., 2020; Sullivan et al., 2021), with evidence indicating that attending private schools is associated with more psychological distress at age 16 for females and predicts it at the age of 42 (Sullivan et al., 2021). Liu and Zhao (2016) found that Chinese youth attending private schools also reported lower LS, experiencing more racial discrimination than their public-school peers.

While research suggests there is no trade-off between academic and wellbeing outcomes (Gutman & Vorhaus, 2012; Lindorff, 2021; Shoshani et al., 2016), a strong focus on academic performance may undermine wellbeing and mental health. A greater emphasis on exams and performance appears to contribute to lower mental health in girls (Giota & Gustafsson, 2017; Högberg et al., 2020; Long et al., 2021; Sonmark et al., 2016) and youth from upper middle-class families in high achieving schools (Cho & Chan, 2020; Ebbert et al., 2019a, 2019b; Heller-Sahlgren, 2018; Luthar et al., 2020). Fear of failure in high-achieving youth from economically prosperous nations has also been linked to lower LS and wellbeing (Borgonovi & Han, 2021; Govorova et al., 2020; Wang, 2021).

### Mental Health and Wellbeing in the United Arab Emirates

Few studies exist relative to youth mental health and wellbeing in the UAE and Gulf region (Alzahrani, 2020; Chan et al., 2021; Lansford et al., 2019; Maalouf et al., 2019), however, a picture is emerging. Across the Eastern Mediterranean Region (EMR), the WHO classification for the Middle East/North Africa, Charara et al., (2017, 2018) estimated that depression and anxiety were the third and ninth leading cause of nonfatal burden of disease. Most nations had higher burdens of mental disorders than global means, particularly for women, with anxiety disorders peaking between 15 and 19 years of age, conduct disorders and ADHD at 10 to 14, and depressive disorders at 40 to 44 years. The increase in burden of mental health was most marked in the Gulf nations, including the UAE. Recently, Chan et al. (2021) calculated pooled prevalence rates for depression (between 26 and 46%) and anxiety (between 17 and 57%) in the Gulf region’s youth, noting their upper limits were above global rates.

A study conducted in the UAE’s city of Al Ain on 600 students between the ages of 12 and 18 found that depressive symptoms were reported in 17% of them (Shah et al., 2020), with rates highest in South Asian expatriates, where one in three reported depressive symptoms, followed by one in five Emirati nationals. Western expatriate students reported half the rates, but all groups had higher than global averages. In another UAE study, Al-Yateem et al., (2020) found that females had double the rate of anxiety and that low to middle SES youth had greater anxiety than those at the higher end. In terms of wellbeing in childhood or adolescence, recent international studies (OECD, 2017, 2019; Marquez & Main, 2020; Marquez & Long, 2020) have provided insights with evidence indicating that between 2015 and 2018, the UAE was among the top third countries where the OLS of 15-year-old adolescents had declined the most.

Potentially related, research on academic outcomes reveal important socio-demographic inequalities in adolescents outcomes in the UAE. For instance, Buckner (2018) found that low-income Emirati nationals had worse academic performance on the 2015 and 2018 PISA exams (OECD, 2019) than their higher income peers, with the influence of SES on academic achievement stronger in the UAE than elsewhere. In addition to the three-year private–public academic performance gap (Marquez et al., 2022a, b, c), there is also a gender gap. Nationally, girls have outperformed boys in all PISA subjects since 2012 and this gap is also larger than anywhere else (OECD, 2019).

The present analysis, the first of its kind, has special relevance and supports the UAE’s dedication to promoting higher wellbeing across the nation. This is especially relevant for national prosperity as difficulties in childhood predict similar issues in adulthood (Auerbach et al., 2018; Johnson et al., 2018; Otto et al., 2021; Trotta et al., 2020). Thus, to lay the groundwork for the present analysis, as well as future studies on how existing inequalities (i.e., wealth, gender) potentially influence wellbeing and mental health, we address the following questions:

1. How does the wellbeing and mental health of adolescents in the UAE differ across the nation’s emirates (geographical regions within the country), socio-demographic groups and school types?
2. Do these inequalities vary across distinct domains of wellbeing and mental health?
3. Is there a trade-off between academic competence and wellbeing and mental health outcomes in the UAE?
4. Did the decline in wellbeing between 2015 and 2018 manifest similar group inequalities?

## Methods

### Participants

The sample included 19,277 15-year-old students who participated in the PISA study 2018 in the UAE. The PISA 2015 sample, used only in Sect. 3.4 (research question 4), included 13,643. Information about the number of participants across each socio-demographic group is reported in Sect. 2.2. For information about the PISA study, see PISA’s technical report (OECD, 2018).

### Measures

Our dependent variables included measures of cognitive subjective/hedonic wellbeing (OLS), affective subjective/hedonic wellbeing (positive affect, and negative affect), psychological/eudaimonic wellbeing (meaning and purpose in life), and mental health (internalizing difficulties). Specifically:

- OLS. Students were asked to rate their satisfaction with life overall on a scale with 0 representing the lowest satisfaction possible and 10, the highest. Data were missing for 9.39% of participants in PISA 2018 (3.98% in PISA 2015).
- Positive affect. Participants were asked how often (never, rarely, sometimes, always) they felt happy, lively, proud, joyful, cheerful. A scale was derived summing up scores from these five items (Cronbach’s alpha 0.8266; 11.38% missing data).
- Negative affect. Students were asked how often (never, rarely, sometimes, always) they felt scared, miserable, afraid, sad, cheerful. A scale was derived summing up scores from these four items (Cronbach’s alpha 0.7912; 11.82% missing data).
- Meaning and purpose in life (Eudaimonic/Psychological well-being). This was measured by asking about the level of agreement (strongly disagree, disagree, agree, strongly agree) with the following: My life has clear meaning or purpose; I have discovered a satisfactory meaning in life; I have a clear sense of what gives meaning to my life. A scale was created by summing up scores (Cronbach’s alpha 0.8611; 8.86% missing data).
- mental health (internalizing difficulties). Participants were asked to report how often over the preceding six months (rarely or never, about every month, about every week, more than once a week, about every day) they had experienced somatic symptoms and symptoms of mental health difficulties: headache, stomach pain, back pain, feeling depressed, irritability of bad temper, feeling nervous, difficulties in getting to sleep, feeling dizzy, feeling anxious. A scale was created by summing up scores in each item (Cronbach’s alpha 0.8957; 16.71% missing data).

Our independent variables included the following socio-demographic groups:

- Gender. This is a binary variable (0 male, 1 female). The sample includes 49% boys and 51% girls in both PISA 2018 and PISA 2015.
- Socio-economic status. PISA’s index of family wealth (see PISA’s technical report: OECD, 2018) was standardized, so that 0 indicates the mean in the UAE and 1, the standard deviation.
- Immigrant background. We created this binary variable by assigning a value of 0 to nationals (i.e., students born in the UAE and whose father and mother were also born in the UAE) and a value of 1 to expatriates (students who were not born in the UAE or whose father or mother was not born in the UAE). In our sample, 44% of participants are nationals and 56% are expatriates (45% and 55% respectively in PISA 2015).
- School sector. We derived a binary variable that assigns a value of 0 if the student is attending a public school (i.e., publicly operated and funded; 40% of participants in PISA 2018, 42% in PISA 2015) and 1 if the student is attending a private school (i.e., privately operated and funded; 60% of participants in PISA 2018, 58% in PISA 2015).
- School curriculum. This is a categorical variable with the following categories: Ministry of Education (MoE, 50.08% in PISA 2018; 49.82% in PISA 2015), British (14.03%; 11.38%), American (13.20%; no data in PISA 2015), Indian (12.09%; 4.76%), Technical and Vocational (TVET, 4.30%; no data in PISA 2015), Other (6.30%; 34.04%). The proportion of students attending private schools in each type of school is: MoE (22.22%; 28.29%), British (96.33%; 100%), American (98.73%; no data in PISA 2015), Indian (100%; 82.04%), TVET (0%; no data in PISA 2015), Other (97.08%; 95.95%).
- Emirate. This is a categorical variable including Abu Dhabi (33.46% in PISA 2018; 25.07% in PISA 2015), Dubai (31.82%; 41.76%), Sharjah (10.98%; 7.30%), Ajman (7.13%; 5.51%), Umm Al Quwain (UAQ, 2.71%; 3.19%), Ras al Khaimah (RAK, 8.56%; 8.33%), Fujairah (5.34%; 8.83%).

### Analytical Approach

To answer our research questions, we conducted linear regression analyses using maximum likelihood estimation. This is the recommended approach to analyse PISA data (OECD, 2009). We also followed recommendations with regards to PISA’s complex design (OECD, 2018) and the need to account for the probability of the student being selected to participate in the study. To do this, we used PISA’s replicate weights. A sequential approach to estimating these linear regression models was used to examine geographic (emirate) differences, socio-demographic (gender, national/expatriate, SES) differences, and differences across types of schools. This approach involved the following steps. For each dependent variable, we ran a series of linear regression models, sequentially introducing each independent variable. For OLS, first, we used the original PISA 0–10 scale first (Table 1 in Sect. 3.1). Next, in Sect. 3.2, in order to compare across different wellbeing and mental health scales, we created a standardized version of the scales on OLS, positive affect, negative affect, meaning and purpose in life, and internalizing difficulties (Table 2 in Sect. 3.2. and Tables 6, 7, 8, 9 and 10 in Appendix 1). Next, in Sect. 3.3, to consider academic competence (i.e. average PISA 2018 score in reading, maths, and science) and its links to wellbeing and mental health, we created an additional model incorporating this variable (Table 3 in Sect. 3.3., and Tables 6, 7, 8, 9 to 10 in Appendix 1). Finally, to estimate changes in OLS between 2015 and 2018 in Sect. 3.4, we used a sample including all the UAE students participating in PISA 2015 and 2018. For each socio-demographic group, we ran a regression model with OLS as the dependent variable and cohort (i.e., the year in which the PISA test was taken) as the only independent variable in the model (Table 5 in Sect. 3.4). OLS was the only wellbeing and mental health variable collected in 2015 and 2018. Levels of missing data were relatively low (see details Sect. 2.2.). We use listwise deletion to deal with missing data, which is common in analyses using this data set (Schirripa et al., 2020; Tsai et al., 2018; van Hek et al., 2018). Analyses were conducted in STATA 15 (StataCorp, 2017).

**Table 1:** Output from linear regression models: socio-demographic differences in OLS from 0 to 10

Variable	Model 1		Model 2		Model 3		Model 4		Model 5		Model 6	
	*B*	*se*	*B*	*se*	*b*	*se*	*B*	*se*	*b*	*se*	*b*	*se*
Constant	6.842***	0.059	7.052***	0.073	7.228***	0.082	7.142***	0.088	7.269***	0.099	7.440***	0.077
Emirate (ref.category: Abu Dhabi): Dubai	-0.123	0.083	-0.130	0.077	-0.072	0.078	-0.101	0.080	0.001	0.087	0.131	0.079
Emirate (ref.category: Abu Dhabi): Sharjah	-0.128	0.172	-0.107	0.159	-0.075	0.156	-0.054	0.160	-0.023	0.155	-0.034	0.122
Emirate (ref.category: Abu Dhabi): Ajman	0.420**	0.133	0.421**	0.141	0.432**	0.142	0.486***	0.140	0.516***	0.153	0.360**	0.135
Emirate (ref.category: Abu Dhabi): UAQ	0.455**	0.157	0.466*	0.184	0.369	0.194	0.373*	0.179	0.311	0.190	0.320	0.191
Emirate (ref.category: Abu Dhabi): RAK	0.652***	0.187	0.645***	0.191	0.581**	0.191	0.587**	0.190	0.517**	0.197	0.387*	0.182
Emirate (ref.category: Abu Dhabi): Fujairah	0.808***	0.172	0.803***	0.187	0.725***	0.189	0.727***	0.187	0.646***	0.193	0.606***	0.176
Female			-0.402***	0.082	-0.422***	0.082	-0.437***	0.083	-0.473***	0.085	-0.462***	0.063
Expatriate					-0.310***	0.067	-0.148*	0.073	0.063	0.076	0.118	0.071
Socio-economic status							0.226***	0.038	0.230***	0.038	0.331***	0.035
Private school									-0.425***	0.095		
School type (ref.category: MoE): British											-1.382***	0.132
School type (ref.category: MoE): American											-0.884***	0.096
School type (ref.category: MoE): Indian											-0.400**	0.129
School type (ref.category: MoE): TVET											-1.656***	0.140
School type (ref.category: MoE): Other											-0.665***	0.139
Observations	17,467		17,467		16,960		16,941		16,326		16,941	

Standard errors (se); ***p < 0.01, **p < 0.05, *p < 0.1

## Results

### Socio-Demographic Differences in Overall Life Satisfaction

The average OLS score of 15-year-old students in the UAE in 2018 was 6.88, but results from Table 1 show significant geographic variation. Compared to Abu Dhabi, OLS was not statistically different in Dubai and Sharjah, but it was higher in the northern, less-urbanized emirates of Ajman, UAQ, RAK and Fujairah. Such differences decrease slightly (and become non-statistically significant for UAQ) after controlling for national/expatriate status, and again after controlling for school sector (public/private) and curriculum, although they remain substantial, ranging from 0.360 points in Ajman to 0.606 points in Fujairah.

Further, females reported almost a half-point lower OLS than males (i.e., -0.462 points lower in the final model 5 controlling for all other socio-demographic variables). Expatriates reported lower OLS than nationals (-0.310 points in model 3), but this gap is half the size after controlling for socio-economic status (-0.148 points in model 4) and becomes non-statistically significant after controlling for school sector and curriculum. Socioeconomic status was positively associated with OLS. In the final model 6 with all the control variables included an increase of 1 standard deviation in the index of socioeconomic status was associated with an increase of 0.331 points in the 0 to 10 OLS scale.

Students attending private schools also reported lower OLS (-0.425 points) than their peers attending public schools after controlling for all socio-demographic characteristics (model 6). Differences across school curriculums were even greater. Compared to schools following the MoE curriculum, OLS was substantially lower in TVET schools (-1.656 points), British schools (-1.382 points), American schools (-0.884 points), schools following other curriculums (-0.665 points), and Indian schools (-0.400 points).

### Socio-Demographic Differences Across Wellbeing and Mental Health Domains

To compare socio-demographic variations across wellbeing and mental health domains, we conducted the same analysis as in Sect. 3.1. for standardized scales of OLS, positive affect, negative affect, meaning and purpose in life, and mental health (internalizing difficulties). A summary of this comparative analysis is presented in Table 2, which shows the results of the final model (i.e., model 5 for public/private school and model 6 for all other independent variables) for each wellbeing and mental health variable. Results for models 1 to 6 for each of these standardized scales is presented in tables 6, 7, 8, 9 to 10 in Appendix 1.

**Table 2:** Output from linear regression analysis (models 5 and 6): socio-demographic differences in OLS, positive affects, negative affects, meaning and purpose in life, and internalizing difficulties (standardized scales)

Variable	OLS		Positive affects		Negative affects		Meaning and purpose in life		Internalizing difficulties	
	*B*	*Se*	*B*	*Se*	*b*	*Se*	*b*	*se*	*b*	*se*
Constant	0.173***	0.027	-0.024	0.025	-0.408***	0.031	0.123***	0.027	0.006	0.035
Emirate (ref.category: Abu Dhabi): Dubai	0.046	0.028	0.050*	0.025	-0.034	0.027	0.032	0.031	-0.009	0.031
Emirate (ref.category: Abu Dhabi): Sharjah	-0.012	0.043	0.063	0.039	-0.017	0.049	0.071	0.043	0.003	0.038
Emirate (ref.category: Abu Dhabi): Ajman	0.126**	0.047	0.141***	0.039	-0.109**	0.037	0.141***	0.040	-0.135***	0.038
Emirate (ref.category: Abu Dhabi): UAQ	0.112	0.067	0.091	0.056	-0.020	0.066	0.096	0.063	-0.048	0.038
Emirate (ref.category: Abu Dhabi): RAK	0.136*	0.064	0.107*	0.049	-0.061	0.057	0.093*	0.047	-0.149**	0.053
Emirate (ref.category: Abu Dhabi): Fujairah	0.213***	0.062	0.179***	0.047	-0.087	0.053	0.263***	0.050	-0.159**	0.058
Female	-0.162***	0.022	0.071***	0.020	0.348***	0.023	0.005	0.022	0.383***	0.023
Expatriate	0.041	0.025	0.063*	0.028	-0.015	0.024	0.089***	0.025	-0.073**	0.028
Socio-economic status	0.117***	0.012	0.148***	0.012	-0.081***	0.014	0.100***	0.013	-0.025	0.013
Private school	-0.149***	0.033	-0.082**	0.028	0.354***	0.036	-0.259***	0.034	-0.177***	0.032
School type (ref.category: MoE): British	-0.486***	0.046	-0.331***	0.044	0.606***	0.057	-0.616***	0.059	-0.118**	0.038
School type (ref.category: MoE): American	-0.311***	0.034	-0.173***	0.033	0.483***	0.034	-0.407***	0.036	-0.138***	0.040
School type (ref.category: MoE): Indian	-0.141**	0.045	0.033	0.040	0.519***	0.035	-0.233***	0.038	-0.497***	0.042
School type (ref.category: MoE): TVET	-0.582***	0.049	-0.308***	0.068	0.462***	0.064	-0.477***	0.058	-0.017	0.050
School type (ref.category: MoE): Other	-0.234***	0.049	-0.124**	0.042	0.513***	0.048	-0.473***	0.048	-0.297***	0.037
Observations	16,941		16,579		16,512		17,034		15,589	

Standard errors (se); ***p < 0.01, **p < 0.05, *p < 0.1

The northern, less-urbanized emirates of Ajman, RAK and Fujairah presented the best wellbeing and mental health outcomes, although for negative affect, this advantage becomes less clear in RAK and Fujairah when controlling for school sector and curriculum. There are no statistically significant differences in wellbeing and mental health between Abu Dhabi and Sharjah, whereas compared to Abu Dhabi, UAQ presented higher meaning and purpose in life, positive affect and OLS (in model 4, 0.143, 0.110, and 0.131 s.d. respectively), but these differences become statistically non-significant after controlling for school sector and school curriculum in models 5 and 6. Finally, compared to Abu Dhabi, Dubai presented lower levels of meaning and purpose in life as well as higher levels of negative affect and internalizing difficulties, although these differences reduce substantially or become non-statistically significant when controlling for school sector and curriculum.

Further, females reported similar levels of meaning and purpose in life to males, slightly higher positive affect (0.071 s.d. in model 6), lower OLS (-0.162 s.d. in model 6), and much higher levels of negative affect (0.348 s.d. in model 6) and internalizing difficulties (0.383 s.d. in model 6). The gender gap in wellbeing and mental health hardly changes after controlling for socio-economic status, immigrant status and school sector/curriculum.

As to SES, the relationship with mental health (internalizing difficulties) was not statistically significant, but links with wellbeing emerged. An increase of one standard deviation in the socioeconomic status scale was associated with higher OLS (0.117 s.d. in model 6), higher meaning and purpose in life (0.100 s.d. in model 6), higher positive affect (0.148 s.d. in model 6), and lower negative affect (-0.081 s.d. in model 6). The size of the association between socio-economic status and these wellbeing variables are similar in models 4 and 5 (i.e., after accounting for school sector), but increase substantially in model 6 (i.e., after accounting for school curriculum).

Compared to national students, expatriate students reported lower wellbeing (in model 3, the gaps are -0.109 s.d. for OLS, 0.226 s.d. for negative affect, -0.067 s.d. for positive affect, and -0.108 s.d. for meaning and purpose in life), but also lower levels of internalizing difficulties (in model 3, -0.196 s.d.). Yet, after controlling for socio-economic status, the wellbeing gaps reduce substantially (and become non-statistically significant in the case of positive affect), and after controlling for school sector and curriculum, they become non-statistically significant for OLS and negative affect, and reverse direction for meaning and purpose in life (0.089 s.d. in model 6) and positive affect (0.063 s.d. in model 6). For internalizing difficulties, the gap remains similar after controlling for SES and reduces substantially when controlling for school sector and curriculum.

Finally, attending private schools is associated with lower OLS (-0.149 s.d.), lower meaning and purpose in life (-0.259 s.d.), higher negative affect (0.354 s.d.) and lower positive affect (-0.082 s.d.), but also lower internalizing difficulties (-0.177 s.d.). Differences across school curriculum are notable. Overall, MoE schools present better wellbeing outcomes compared to any other type of school (gaps are especially large for negative affect and meaning and purpose in life) and somewhat worse outcomes for internalizing difficulties (although differences are not statistically significant for TVET). In terms of wellbeing, British schools and TVET schools present the poorest outcomes, although these differences are less clear for negative affect. In terms of internalizing difficulties, Indian schools present better outcomes than any other type.

### Wellbeing and Mental Health and Academic Outcomes: Is there a Trade-Off?

Considering the results reported in Sect. 3.2 with findings on academic competence in the UAE (scores in PISA 2018) reported in Marquez et al. (2022b), some groups of students who do better academically seem to present worse wellbeing outcomes. To illustrate, Table 3 presents PISA 2018 score inequalities (average for reading, maths, and science) across our groups of interest. The average PISA score across the UAE was 434 points, and the s.d. 100 points. Table 3 shows that academic competence is highest in Dubai (484 points on average) and lowest in the northern emirates (around 400 points); higher among females (449 points) compared to males (418 points); higher among expatriate students (476 points) compared to national students (389 points); and higher in private schools (469 points) versus public schools (379 points). Academic competence is highest in British schools (510 points), followed by Indian schools (494 points), Other schools (484 points), American schools (445 points), TVET schools (397), and MoE schools (388 points).

**Table 3:** Group differences in the average PISA 2018 score (readings, maths, science)

Variable	Average PISA 2018 score (reading, maths, science)	Difference		
		*b*	*se*	Group comparison
Abu Dhabi	410			
Dubai	484	74***	7.1	Abu Dhabi—Dubai
Sharjah	442	32***	8.6	Abu Dhabi—Sharjah
Ajman	400	-10	7.8	Abu Dhabi—Ajman
UAQ	397	-13	10.1	Abu Dhabi—UAQ
RAK	402	-8	7.2	Abu Dhabi—RAK
Fujairah	398	-12	9.0	Abu Dhabi—Fujairah
Male	418			
Female	449	31***	6.1	Male—Female
National	389			
Expatriate	476	87***	3.5	National—Expatriate
Socio-economic status	-	-1	1.5	Socio-economic status
Public school	379			
Private school	469	90***	4.8	Public school—Private school
MoE	388			
British	510	122***	5.6	MoE—British
American	445	57***	8.6	MoE—American
Indian	494	106***	6.8	MoE—Indian
TVET	397	9	11.6	MoE—TVET
Other	484	96***	13.7	MoE—Other

Standard errors (se); ***p < 0.01, **p < 0.05, *p < 0.1

Given that some groups present higher academic competence but lower wellbeing outcomes (e.g. females, expatriates, students from Dubai, students attending private schools, especially British schools, etc.), we are compelled to ask whether there is a trade-off between academic competence and wellbeing in the UAE? We explored this question estimating an additional model 7 (Tables 6, 7, 8, 9 to 10 in Appendix 1). Model 7 contains the same independent variables as model 6 but adds academic competence (i.e. average PISA 2018 score in reading, maths, and science). The results are summarized in Table 4.

**Table 4:** Output from linear regression analysis (model 7): socio-demographic differences in OLS, positive affects, negative affects, meaning and purpose in life, and internalizing difficulties (standardized scales), after adding academic competence as an additional independent variable

Variable	OLS		Positive affects		Negative affects		Meaning and purpose in life		Internalizing difficulties	
	*B*	*Se*	*B*	*Se*	*b*	*Se*	*b*	*se*	*b*	*se*
Constant	0.189***	0.028	0.026***	0.025	0.439	0.032	0.125	0.027	0.010	0.034
Emirate (ref.category: Abu Dhabi): Dubai	0.039	0.028	0.028	0.026	-0.021	0.028	0.032	0.031	0.003	0.030
Emirate (ref.category: Abu Dhabi): Sharjah	-0.014	0.043	0.055	0.039	-0.012	0.048	0.071	0.043	-0.006	0.038
Emirate (ref.category: Abu Dhabi): Ajman	0.129*	0.047	0.147***	0.038	-0.112**	0.036	0.141***	0.040	0.137***	0.038
Emirate (ref.category: Abu Dhabi): UAQ	0.112	0.068	0.089	0.057	-0.019	0.065	0.096	0.063	0.047	0.037
Emirate (ref.category: Abu Dhabi): RAK	0.135*	0.064	0.102*	0.048	-0.058	0.057	0.092*	0.046	0.147**	0.053
Emirate (ref.category: Abu Dhabi): Fujairah	0.212**	0.062	0.175***	0.045	-0.085	0.053	0.263***	0.050	0.158**	0.058
Female	-0.168***	0.022	0.054*	0.020	0.359***	0.023	0.005	0.022	-0.388***	0.023
Expatriate	0.029	0.026	0.023	0.029	0.010	0.025	0.088***	0.025	0.061*	0.027
Socio-economic status	0.114***	0.012	0.142***	0.012	-0.077***	0.014	0.100***	0.013	0.022	0.013
Private school										
School type (ref.category: MoE): British	-0.505***	0.046	-0.391***	0.043	0.643***	0.056	-0.618***	0.058	-0.099*	0.039
School type (ref.category: MoE): American	-0.321***	0.034	-0.205***	0.035	0.503***	0.035	-0.408***	0.036	-0.128**	0.041
School type (ref.category: MoE): Indian	-0.157**	0.046	-0.018	0.040	0.551***	0.035	-0.234***	0.039	-0.481***	0.043
School type (ref.category: MoE): TVET	-0.588***	0.050	-0.326***	0.063	0.472***	0.062	-0.478***	0.058	-0.012	0.049
School type (ref.category: MoE): Other	-0.249***	0.051	-0.173***	0.046	0.544***	0.051	-0.474***	0.050	-0.281***	0.038
Academic competence (Mean PISA score)	0.024*	0.012	0.075***	0.013	-0.047***	0.012	0.002	0.014	0.024	0.012
Observations	16,941		16,941		16,512		17,034		15,589	

Standard errors (se); ***p < 0.01, **p < 0.05, *p < 0.1

Results show that an increase of 100 points (1 s.d.) on average in the three core PISA subjects (reading, maths, and science) is associated with small increases in OLS (0.024 s.d.) and positive affect (0.075 s.d.), as well as a small decrease in negative affect (-0.047 s.d.). Effects are not statistically significant for meaning and purpose in life and internalizing difficulties. A comparisson between results in models 6 in Table 2 and models 7 in Table 4 also show that, when adding academic competence to the models, the other estimates barely change in most cases, although some small changes are observed in the positive affect and negative affect models for school type and emirate.

### Socio-Demographic Differences in Changes to Overall Life Satisfaction between 2015 and 2018

In view of socio-demographic differences in levels of wellbeing and mental health in 2018, we explored whether declines in student OLS between 2015 and 2018 also followed different patterns across distinct socio-demographic groups. OLS was the only wellbeing variable collected in both PISA waves. Table 5 shows how OLS changed on average for each socio-demographic group between 2015 and 2018. On average across the UAE, OLS declined by 0.42 points on the 0 to 10 scale, but this decline was more pronounced for some groups. The decline was largest in Sharjah (-0.55 points) and Abu Dhabi (-0.50 points), followed by RAK (-0.42 points) and Dubai (-0.37 points). Changes were not statistically significant elsewhere.

**Table 5:** Output from linear regression models: socio-demographic differences in changes in OLS (from 0 to 10) between 2015 and 2018

		Satisfaction with life as a whole (0–10 LS scale)				
					Difference	
		2015	2018	*B*		s.e
UAE		7.31	6.88	-0.42	***	0.05
Emirate	Abu Dhabi	7.34	6.84	-0.50	***	0.08
	Dubai	7.09	6.72	-0.37	***	0.06
	Sharjah	7.27	6.71	-0.55	**	0.20
	Ajman	7.45	7.26	-0.18		0.19
	UAQ	7.32	7.30	-0.03		0.21
	RAK	7.91	7.49	-0.42	***	0.12
	Fujairah	7.66	7.65	-0.01		0.14
Gender	Male	7.45	7.10	-0.35	***	0.07
	Female	7.18	6.69	-0.49	***	0.07
Immigrant status	National	7.60	7.12	-0.48	***	0.07
	Expatriate	7.12	6.71	-0.41	***	0.07
SES	Lowest SES	6.68	6.60	-0.09		0.13
	Mid-low SES	6.90	6.71	-0.19		0.11
	Mid SES	6.87	6.87	-0.01		0.11
	Mid-high SES	7.26	6.74	-0.51	***	0.11
	Highest SES	7.64	7.07	-0.58	***	0.07
School sector	Public school	7.65	7.22	-0.43	***	0.06
	Private school	7.12	6.66	-0.45	***	0.08
School curriculum	MoE	7.44	7.34	-0.09		0.05
	British	7.03	5.97	-1.06	***	0.16
	American					
	Indian	7.30	6.83	-0.47	*	0.20
	TVET					
	Other	7.24	6.79	-0.45	***	0.12

The decline in OLS was also greater among females (-0.49 points) compared to males (-0.35 points), nationals (-0.48 points) compared to expatriates (-0.41 points), and students of higher socio-economic status. With respect to the latter, differences were not statistically significant for those in the first, second, and third quintile, but were substantial for those in the fourth (-0.51 points) and fifth quintile (-0.58 points).

Finally, the decline in OLS was similar in size in public schools (-0.43 points) and private schools (-0.45 points), but differences across schools following distinct curriculums were indeed substantial. Changes were not statistically significant in MoE schools, but the decline in OLS was very large in British schools (-1.06 points). In Indian schools (-0.47 points) and schools following other curriculums (-0.45 points), this decline was slightly above the national average. Information about American and TVET schools was not included in PISA 2015, so no comparisons were possible for these schools.

## Discussion

### Findings

The present study revealed important geographic, socio-demographic, and school type differences in trends and levels of adolescent wellbeing and mental health in the UAE. First, the northern emirates of Ajman, RAK and Fujairah presented better wellbeing and mental health outcomes, although these differences became somewhat smaller when controlling for school sector and school type. In these emirates, there are fewer expatriate residents, private schools, and schools overall (Sheikh Saud bin Saqr Al Qasimi Foundation for Policy Research, 2020); hence, it may be that schools suffer from fewer social comparisons to other schools like those ranked in bigger urban centres. They may have a more homogenous student body and be able to instil a greater sense of belonging and/or identity. Their lead may also reflect the geographical advantage of rural areas to mid-size cities (i.e., Buttazzoni et al., 2022; Murphy et al., 2020; Tomyn et al., 2017), the impact of lower urban density or quality of place and neighbourhood, or greater access to nature (Carmona, 2019; Gorski-Steiner et al., 2022; Rentfrow, 2018; Silva et al., 2016; Vanaken & Danckaerts, 2018; Ward et al., 2016).

In contrast, the emirate of Dubai presented notably lower scores for meaning and purpose, higher scores for negative affect and the greatest mental health issues of all the emirates. This is a striking finding given that it is the only emirate to officially endorse school wellbeing (OECD, 2021). Its education authority has promoted wellbeing as the cornerstone of its efforts and is an item against which schools are inspected and classed (see OECD, 2021). Perhaps its highly competitive, performance focused and publicized school ranking scheme which allows for parents to make informed choices as well as school fees to be raised accordingly, has created unintended effects on wellbeing (Sheikh Saud bin Saqr Al Qasimi Foundation for Policy Research, 2020; El-Sholkamyl & Al-Saleh, 2017; OECD, 2021). Still, the emirate has the highest number of students, private schools and diversity of all, making its challenges greater. It also has higher than average rates of bullying (KHDA, 2021; OECD, 2021), which may be driving the higher rates of mental health issues. Ranking best for academic performance, student anxiety and parental pressure is higher than in other OECD countries and may also contribute (OECD, 2021). Taking stronger leadership in guiding wellbeing and mental health policy and programming may be beyond its legislative reach and account for the disparity between its endorsement of wellbeing versus implementation.

Females also reported more mental health issues, lower OLS, higher negative affect and somewhat higher positive affect. These findings are consistent with prior studies (Al-Yateem et al., 2020; Bradshaw et al., 2013; Campbell et al., 2021; Charara et al., 2017, 2018; Cyranowski et al., 2000; Hamama & Hamama-Raz, 2021; Kaye-Tzadok et al., 2017). However, that no gender differences were observed for meaning and purpose in life is inconsistent with findings by Campbell et al. (2021), who find that in most countries, meaning and purpose in life among 15-year-old adolescents is higher in males. Gender differences were larger with regards to negative affect and internalizing difficulties compared to positive affect and OLS.

Our findings also confirm a long-standing truism that for every increase in SES gradient, there are parallel increases in wellbeing (Chzhen et al., 2016; Elgar et al., 2017; Knies, 2022; Marquez, 2021; Patalay & Fitzsimons, 2018; Zaborskis et al., 2019a, b). However, higher SES was associated with more mental health difficulties, which contradicts findings from previous literature reviews (Reiss, 2013) and meta-analyses (Peverill et al., 2021; Reiss et al., 2019), and seem to suggest that as resources stemming from greater income can buy experiences and time with which to enjoy life and find it satisfying, the same cannot always be said of those same resources defending against mental health issues, like depression, anxiety, etc. (Killingsworth, 2021), which present a particularly high incidence (above global rates) in the GCC region (Chan et al., 2021).

Findings around the national/expatriate gaps in wellbeing and mental health are compelling. First, expatriates reported lower wellbeing, but also fewer mental health issues than nationals. Second, after controlling for SES, the gap in wellbeing reduces, but the mental health gap remains. The fact that the national/expatriate gap in wellbeing, but not mental health, is partly explained by differences in SES aligns with the point discussed above that SES (material well-being) tends to be associated with better wellbeing outcomes but may not necessarily be associated with better mental health (see Mogilner et al., 2018), especially in a context of high incidence of population levels of mental health difficulties (Chan et al., 2021). After controlling for school type (i.e., school sector, curriculum), the wellbeing gaps almost disappear for OLS and negative affect, and reverse direction for meaning and purpose in life. The mental health gap reduces as well. This suggests that schools indeed play a role in wellbeing and mental health as they explain part of the gaps across the emirates and a large proportion of the national/expatriate gaps.

Compared to private schools, public schools present higher wellbeing but poorer mental health outcomes, and this holds true after controlling for socio-demographic characteristics. Similarly, the poorest mental health levels are observed in MoE and TVET schools, and the best in Indian schools. The latter finding contrasts with a previous UAE study highlighting higher rates of internalizing disorders (i.e., depression) in South Asian students in the city of Al Ain (Shah et al., 2020). The lowest wellbeing is observed in TVET and British schools, and the highest in MoE schools, followed by Indian schools. American schools and schools following other curriculums are somewhere in the middle in terms of wellbeing and mental health relative to other types of schools. The low wellbeing of TVET schools may be due to their poor public image and status as students and their parents consider them options for low performers (Khan et al., 2018).

Why mental health issues are higher in lower performing schools (here, MoE schools) suggests school, community, or cultural factors might play a role. Mental health issues may be unidentified and hence, overly represented (Lansford et al., 2019; Maalouf et al., 2019). Alternatively, they may be better tolerated. In Western cultures, there is pressure to reduce negative emotional experiences, whereas in many Eastern cultures, distress is accepted and less “psychologized” (e.g., Eloul et al., 2009; Huang et al., 2020). Thus, mental health issues are construed as an inconvenience and reflect a dialectical emotional state where they co-exist with and are less impactful on wellbeing than they might in other cultural contexts (Lomas, 2016). Similarly, it may be a third force that is not being captured in the Western measures being used in PISA (although they have been regionally validated); that is, the unity between mind, body, community, and spiritual aspects of the region (Joshanloo et al., 2021), which has not yet been captured or investigated by education authorities either (OECD, 2021). School factors that produce low academic performance may also fuel mental health concerns, especially as one drives the other (Agnafors et al., 2021; Deighton et al., 2018; Pagerols et al., 2022; Riglin et al., 2014). Alternatively, issues identified in UAE public schools, i.e., non-national teachers, low status of teaching, low teacher wellbeing, few resources, and little parent support (Marquez et al., 2022b) may contribute.

In terms of wellbeing, how can the high wellbeing observed in low-performing MoE schools be explained? And why is wellbeing so low in high-performing British schools? This question deserves further exploration; however, our study suggests that this is not related to academic outcomes. We found no evidence for a trade-off between academic and wellbeing and mental health outcomes. In fact, we found a small positive association with subjective wellbeing, especially with the affective component. Higher academic competence was associated with higher OLS, higher positive affect, and lower negative affect –although for OLS, the size of the effect was rather small and could potentially be the result of a method effect. Overall, this finding is consistent with previous research reporting (Gutman & Vorhaus, 2012; Lindorff, 2021; Shoshani et al., 2016). As happier students make better learners, schools and education authorities should promote both wellbeing and academic outcomes (Taylor et al., 2022).

To promote adolescent wellbeing, more research is needed to understand what policy-relevant factors influence it. Research highlights factors such as high-stakes testing, fear of failure, performance anxiety, and sense of belonging to school (Borgonovi & Han, 2021; Cho & Chan, 2020; Högberg et al., 2020; Long et al., 2021; Marquez & Main, 2020; Sonmark et al., 2016; Wang, 2021) are associated with lower wellbeing, which might explain some of our results. Further, British schools in the UAE achieve the best education inspection ratings, which are largely defined in terms of academic outcomes, and consequently demand the highest school fees (El-Sholkamyl & Al-Saleh, 2017). However, our study shows that they also present the worst wellbeing outcomes and relatively poor mental health outcomes. To provide the right incentives for wellbeing and mental health promotion, we recommend that education authorities incorporate wellbeing and mental health outcomes in their school inspections and that these be made transparent in the rankings. This is vital given the null or positive association between wellbeing and academic outcomes described and the predictive association between wellbeing and mental health in adolescence and later life outcomes (Cavioni et al., 2021; DeNeve & Oswald, 2012; Goodman et al., 2015; Guzmán et al., 2020; Kansky et al., 2016; Richards & Huppert, 2011).

Finally, understanding trends can help better understand geographic, socio-demographic and school type differences in adolescents’ wellbeing. As noted in Marquez and Long (2020), 15-year-old adolescents’ OLS declined in the UAE between 2015 and 2018, as it did in most countries, and the UAE was among the top third of countries presenting the largest decline. Yet, in the present study we found that this decline was larger among females, national students, students of higher SES and in British schools, as well as in the emirates of Sharjah, Abu Dhabi, RAK and Dubai. This may be explained by rising mental health issues, which serve to drag OLS scores downward (Badri et al., 2022; Lombardo et al., 2018). Declines in youth wellbeing have been especially pronounced in countries that are more egalitarian, with higher GDP per capita, and where concurrent increases in academic pressure are observed (Borgonovi & Han, 2021; Cosma et al., 2020). Furthermore, in a recent study, Marquez et al. (2022c) examined factors associated with declines in adolescent OLS between 2015 and 2018 in the UK, the United States, France, Ireland and Japan, finding that this decline may be largely due to changes in school well-being and, to a lesser extent, material well-being and the use of Information and Communication Technologies. They also found differences by gender and across countries, notably between Western nations and Japan. This highlights again the importance of national studies on adolescent wellbeing and mental health that account for the specific context and socio-demographic profile of the country as levels and changes over time –and the factors associated with these- not only differ across countries but potentially also across different socio-demographic groups (e.g. gender, ethnicity, migrant status, etc.) within nations.

### Limitations and Future Directions

We analyzed pre-Covid-19 data. The next PISA edition (2022) will be revelatory for the status of youth wellbeing and mental health, especially as the effects of the pandemic will linger beyond its end (Gavin et al., 2021; Racine et al., 2021; UNICEF, 2021). Also, due to the cross-sectional nature of the data and the research design used, causality cannot be established. Moreover, although levels of missing data were generally low for all the wellbeing outcomes, these were somewhat higher for mental health symptoms of internalising difficulties (see Sect. 2.2.); thus, the results involving this variable should be interpreted with more caution. Another important limitation to keep in mind refers to the validity of the Western-derived measures of wellbeing included in PISA in a non-Western nation such as the UAE where adolescents may understand and respond to questions on their wellbeing in substantially different ways compared to their Western peers due to cultural and linguistic issues. Although the body of research assessing the cross-cultural validity of adolescent wellbeing measures is growing (Casas & Gonzalez-Carrasco, 2021), more research is needed to examine the validity of these specific measures of adolescent wellbeing in the UAE. Overall, while most of our findings coincided with the literature, the picture within the UAE is complex and confirms the need for continued broad-based assessments of this sort, using a range of measures to understand the wellbeing and mental health landscape for young people in the country. This is especially the case in the public-school students, which tend to be mostly nationals, where concurrent mental health issues and high wellbeing operate together and create a student profile worth further investigation, in addition to confirming the two-continua model of wellbeing (Keyes, 2005). We finally echo the OECD’s (2021) recommendation for education authorities to share their data. The KHDA collects longitudinal student wellbeing data in a highly granular form, which can be used to understand what is driving school, familial and community factors that support wellbeing and mental health in the emirate and nation. However, this has not been made public, belabouring efforts and delaying progress.

### Conclusion

In adolescence, wellbeing and mental health should not be considered less important than academic outcomes given their capacity to predict important outcomes later in life, including academic outcomes (Cavioni et al., 2021; DeNeve & Oswald, 2012; Goodman et al., 2015; Guzmán et al., 2020; Kansky et al., 2016; Richards & Huppert, 2011). This stance was further supported by our finding that in the UAE, students who reported greater wellbeing also performed better academically. Consequently, schools, parents and relevant stakeholders should take students wellbeing and mental health seriously. Our findings on geographic, socio-demographic and school type inequalities in adolescent wellbeing and mental health help the identification of the more vulnerable groups in the UAE and provide a baseline against which future changes can be measured. Education authorities at the national and emirate level must invest more heavily in public education to address academic performance, while concurrently addressing mental health across all schools, as well as improving wellbeing outcomes uniformly. These aims will ensure that the ability of young people to effectively make a living, as well as live happy, satisfactory lives, is maximized.

## Appendix

Tables 6, 7, 8, 9 and 10

**Table 6:** Output from linear regression models: socio-demographic differences in OLS (standardized scale)

Variable	Model 1		Model 2		Model 3		Model 4		Model 5		Model 6		Model 7	
	*b*	*se*	*b*	*se*	*b*	*se*	*b*	*se*	*b*	*se*	*b*	*se*	*B*	*se*
Constant	-0.037	0.021	0.037	0.026	0.098***	0.029	0.068*	0.031	0.113**	0.035	0.173***	0.027	0.189***	0.028
Emirate (ref.category: Abu Dhabi): Dubai	-0.043	0.029	-0.046	0.027	-0.025	0.027	-0.036	0.028	0.000	0.031	0.046	0.028	0.039	0.028
Emirate (ref.category: Abu Dhabi): Sharjah	-0.045	0.060	-0.038	0.056	-0.026	0.055	-0.019	0.056	-0.008	0.055	-0.012	0.043	-0.014	0.043
Emirate (ref.category: Abu Dhabi): Ajman	0.148**	0.047	0.148**	0.049	0.152**	0.050	0.171***	0.049	0.181***	0.054	0.126**	0.047	0.129*	0.047
Emirate (ref.category: Abu Dhabi): UAQ	0.160**	0.055	0.164*	0.065	0.130	0.068	0.131*	0.063	0.109	0.067	0.112	0.067	0.112	0.068
Emirate (ref.category: Abu Dhabi): RAK	0.229***	0.066	0.227***	0.067	0.204**	0.067	0.206**	0.067	0.182**	0.069	0.136*	0.064	0.135*	0.064
Emirate (ref.category: Abu Dhabi): Fujairah	0.284***	0.060	0.282***	0.066	0.255***	0.066	0.256***	0.066	0.227***	0.068	0.213***	0.062	0.212**	0.062
Female			-0.141***	0.029	-0.148***	0.029	-0.154***	0.029	-0.166***	0.030	-0.162***	0.022	-0.168***	0.022
Expatriate					-0.109***	0.024	-0.052*	0.026	0.022	0.027	0.041	0.025	0.029	0.026
Socio-economic status							0.079***	0.013	0.081***	0.014	0.117***	0.012	0.114***	0.012
Private school									-0.149***	0.033				
School type (ref.category: MoE): British											-0.486***	0.046	-0.505***	0.046
School type (ref.category: MoE): American											-0.311***	0.034	-0.321***	0.034
School type (ref.category: MoE): Indian											-0.141**	0.045	-0.157**	0.046
School type (ref.category: MoE): TVET											-0.582***	0.049	-0.588***	0.050
School type (ref.category: MoE): Other											-0.234***	0.049	-0.249***	0.051
Academic competence (Mean PISA score)													0.024*	0.012
Observations	17,467		17,467		16,960		16,941		16,326		16,941		16,941	

Standard errors (se); ***p < 0.01, **p < 0.05, *p < 0.1

**Table 7:** Output from linear regression models: socio-demographic differences in positive affects (standardized scale)

Variable	Model 1		Model 2		Model 3		Model 4		Model 5		Model 6		Model 7	
	*b*	*se*	*b*	*se*	*b*	*se*	*b*	*se*	*b*	*se*	*b*	*se*	*b*	*Se*
Constant	-0.034	0.019	-0.087***	0.024	-0.031	0.026	-0.077**	0.028	-0.053	0.031	-0.024	0.025	0.026***	0.025
Emirate (ref.category: Abu Dhabi): Dubai	0.013	0.026	0.015	0.026	0.021	0.026	0.007	0.026	0.033	0.028	0.050*	0.025	0.028	0.026
Emirate (ref.category: Abu Dhabi): Sharjah	0.041	0.046	0.036	0.048	0.049	0.048	0.058	0.050	0.065	0.049	0.063	0.039	0.055	0.039
Emirate (ref.category: Abu Dhabi): Ajman	0.162***	0.044	0.161***	0.042	0.151***	0.043	0.179***	0.041	0.193***	0.042	0.141***	0.039	0.147***	0.038
Emirate (ref.category: Abu Dhabi): UAQ	0.123	0.069	0.120*	0.058	0.105	0.058	0.110*	0.051	0.098	0.053	0.091	0.056	0.089	0.057
Emirate (ref.category: Abu Dhabi): RAK	0.166**	0.054	0.168**	0.052	0.149**	0.051	0.154**	0.050	0.139**	0.052	0.107*	0.049	0.102*	0.048
Emirate (ref.category: Abu Dhabi): Fujairah	0.217***	0.057	0.219***	0.051	0.202***	0.051	0.204***	0.049	0.189***	0.050	0.179***	0.047	0.175***	0.045
Female			0.098***	0.025	0.079**	0.025	0.071**	0.025	0.066*	0.026	0.071***	0.020	0.054*	0.020
Expatriate					-0.067**	0.022	0.017	0.025	0.056*	0.026	0.063*	0.028	0.023	0.029
Socio-economic status							0.119***	0.012	0.121***	0.012	0.148***	0.012	0.142***	0.012
Private school									-0.082**	0.028				
School type (ref.category: MoE): British											-0.331***	0.044	-0.391***	0.043
School type (ref.category: MoE): American											-0.173***	0.033	-0.205***	0.035
School type (ref.category: MoE): Indian											0.033	0.040	-0.018	0.040
School type (ref.category: MoE): TVET											-0.308***	0.068	-0.326***	0.063
School type (ref.category: MoE): Other											-0.124**	0.042	-0.173***	0.046
Academic competence (Mean PISA score)													0.075***	0.013
Observations	17,083		16,593		16,579		15,980		16,579		16,579		16,941	

Standard errors (se); ***p < 0.01, **p < 0.05, *p < 0.1

**Table 8:** Output from linear regression models: socio-demographic differences in negative affects (standardized scale)

Variable	Model 1		Model 2		Model 3		Model 4		Model 5		Model 6		Model 7	
	*b*	*se*	*b*	*se*	*b*	*se*	*b*	*se*	*b*	*se*	*b*	*se*	*B*	*se*
Constant	-0.006	0.024	-0.178***	0.030	-0.299***	0.033	-0.280***	0.035	-0.378***	0.038	-0.408***	0.031	0.439	0.032
Emirate (ref.category: Abu Dhabi): Dubai	0.158***	0.038	0.165***	0.034	0.115***	0.032	0.121***	0.033	0.047	0.033	-0.034	0.027	-0.021	0.028
Emirate (ref.category: Abu Dhabi): Sharjah	0.066	0.081	0.050	0.068	0.017	0.065	0.013	0.066	-0.014	0.060	-0.017	0.049	-0.012	0.048
Emirate (ref.category: Abu Dhabi): Ajman	-0.127**	0.048	-0.128**	0.047	-0.141**	0.048	-0.153**	0.048	-0.173***	0.052	-0.109**	0.037	-0.112**	0.036
Emirate (ref.category: Abu Dhabi): UAQ	-0.098	0.083	-0.109	0.077	-0.065	0.078	-0.066	0.077	-0.009	0.081	-0.020	0.066	-0.019	0.065
Emirate (ref.category: Abu Dhabi): RAK	-0.204***	0.060	-0.197**	0.062	-0.143*	0.060	-0.144*	0.059	-0.083	0.062	-0.061	0.057	-0.058	0.057
Emirate (ref.category: Abu Dhabi): Fujairah	-0.208**	0.066	-0.201***	0.056	-0.143**	0.055	-0.143**	0.054	-0.078	0.054	-0.087	0.053	-0.085	0.053
Female			0.324***	0.036	0.332***	0.034	0.335***	0.033	0.347***	0.033	0.348***	0.023	0.359***	0.023
Expatriate					0.226***	0.025	0.191***	0.028	0.011	0.029	-0.015	0.024	0.010	0.025
Socio-economic status							-0.051**	0.016	-0.052**	0.016	-0.081***	0.014	-0.077***	0.014
Private school									0.354***	0.036				
School type (ref.category: MoE): British											0.606***	0.057	0.643***	0.056
School type (ref.category: MoE): American											0.483***	0.034	0.503***	0.035
School type (ref.category: MoE): Indian											0.519***	0.035	0.551***	0.035
School type (ref.category: MoE): TVET											0.462***	0.064	0.472***	0.062
School type (ref.category: MoE): Other											0.513***	0.048	0.544***	0.051
Academic competence (Mean PISA score)													-0.047***	0.012
Observations	16,999		16,999		16,524		16,512		15,910		16,512		16,512	

Standard errors (se); ***p < 0.01, **p < 0.05, *p < 0.1

**Table 9:** Output from linear regression models: socio-demographic differences in meaning and purpose in life (standardized scale)

Variable	Model 1		Model 2		Model 3		Model 4		Model 5		Model 6		Model 7	
	*b*	*se*	*b*	*se*	*b*	*se*	*b*	*se*	*b*	*se*	*b*	*se*	*b*	*se*
Constant	-0.023	0.019	-0.039	0.025	0.032	0.030	0.010	0.032	0.079*	0.036	0.123***	0.027	0.125	0.027
Emirate (ref.category: Abu Dhabi): Dubai	-0.100**	0.033	-0.099**	0.033	-0.083*	0.033	-0.090**	0.034	-0.021	0.035	0.032	0.031	0.032	0.031
Emirate (ref.category: Abu Dhabi): Sharjah	0.031	0.065	0.029	0.065	0.048	0.064	0.052	0.065	0.073	0.061	0.071	0.043	0.071	0.043
Emirate (ref.category: Abu Dhabi): Ajman	0.184***	0.046	0.184***	0.045	0.187***	0.047	0.200***	0.046	0.229***	0.050	0.141***	0.040	0.141***	0.040
Emirate (ref.category: Abu Dhabi): UAQ	0.165*	0.069	0.164*	0.066	0.140*	0.064	0.143*	0.062	0.103	0.067	0.096	0.063	0.096	0.063
Emirate (ref.category: Abu Dhabi): RAK	0.203***	0.051	0.204***	0.050	0.173***	0.050	0.176***	0.049	0.139**	0.051	0.093*	0.047	0.092*	0.046
Emirate (ref.category: Abu Dhabi): Fujairah	0.349***	0.060	0.350***	0.059	0.317***	0.059	0.318***	0.058	0.277***	0.057	0.263***	0.050	0.263***	0.050
Female			0.030	0.034	0.018	0.034	0.014	0.034	0.004	0.034	0.005	0.022	0.005	0.022
Expatriate					-0.108***	0.026	-0.068*	0.029	0.065*	0.029	0.089***	0.025	0.088***	0.025
Socio-economic status							0.057***	0.015	0.059***	0.015	0.100***	0.013	0.100***	0.013
Private school									-0.259***	0.034				
School type (ref.category: MoE): British											-0.616***	0.059	-0.618***	0.058
School type (ref.category: MoE): American											-0.407***	0.036	-0.408***	0.036
School type (ref.category: MoE): Indian											-0.233***	0.038	-0.234***	0.039
School type (ref.category: MoE): TVET											-0.477***	0.058	-0.478***	0.058
School type (ref.category: MoE): Other											-0.473***	0.048	-0.474***	0.050
Academic competence (Mean PISA score)													0.002	0.014
Observations	17,570		17,570		17,046		17,034		16,415		17,034		17,034	

Standard errors (se); ***p < 0.01, **p < 0.05, *p < 0.1

**Table 10:** Output from linear regression models: socio-demographic differences in internalizing difficulties (standardized scale)

Variable	Model 1		Model 2		Model 3		Model 4		Model 5		Model 6		Model 7	
	*b*	*se*	*b*	*se*	*b*	*se*	*b*	*se*	*b*	*se*	*b*	*se*	*b*	*se*
Constant	0.075*	0.032	-0.135***	0.031	-0.031	0.034	-0.028	0.034	0.024	0.038	0.006	0.035	0.010	0.034
Emirate (ref.category: Abu Dhabi): Dubai	-0.139***	0.041	-0.130***	0.037	-0.087*	0.035	-0.086*	0.035	-0.055	0.036	-0.009	0.031	-0.003	0.030
Emirate (ref.category: Abu Dhabi): Sharjah	-0.028	0.051	-0.051	0.043	-0.019	0.041	-0.018	0.041	-0.008	0.041	0.003	0.038	0.006	0.038
Emirate (ref.category: Abu Dhabi): Ajman	-0.144**	0.053	-0.149**	0.045	-0.142***	0.041	-0.144***	0.041	-0.131**	0.043	-0.135***	0.038	-0.137***	0.038
Emirate (ref.category: Abu Dhabi): UAQ	0.027	0.076	0.004	0.039	-0.038	0.039	-0.038	0.039	-0.072	0.043	-0.048	0.038	-0.047	0.037
Emirate (ref.category: Abu Dhabi): RAK	-0.107	0.062	-0.099	0.056	-0.146**	0.055	-0.145**	0.055	-0.194***	0.057	-0.149**	0.053	-0.147**	0.053
Emirate (ref.category: Abu Dhabi): Fujairah	-0.100	0.069	-0.096	0.060	-0.155*	0.062	-0.153*	0.062	-0.188**	0.061	-0.159**	0.058	-0.158**	0.058
Female			0.395***	0.030	0.390***	0.028	0.390***	0.028	0.387***	0.029	0.383***	0.023	0.388***	0.023
Expatriate					-0.196***	0.027	-0.202***	0.026	-0.111***	0.029	-0.073**	0.028	-0.061*	0.027
Socio-economic status							-0.008	0.013	-0.010	0.014	-0.025	0.013	-0.022	0.013
Private school									-0.177***	0.032				
School type (ref.category: MoE): British											-0.118**	0.038	-0.099*	0.039
School type (ref.category: MoE): American											-0.138***	0.040	-0.128**	0.041
School type (ref.category: MoE): Indian											-0.497***	0.042	-0.481***	0.043
School type (ref.category: MoE): TVET											-0.017	0.050	-0.012	0.049
School type (ref.category: MoE): Other											-0.297***	0.037	-0.281***	0.038
Academic competence (Mean PISA score)													0.024	0.012
Observations	16,056		16,056		15,601		15,589		16,326		15,021		15,589	

Standard errors (se); ***p < 0.01, **p < 0.05, *p < 0.1
